# Case Report: Perioperative application of trastuzumab deruxtecan in *HER2* exon 20 mutation-positive non-small cell lung cancer

**DOI:** 10.3389/fphar.2025.1629854

**Published:** 2026-01-07

**Authors:** Wenzhu Li, Zhenzhen Xiao, Jiaqi Yang, Lirong Liu, Xiaoshu Chai, Haibo Zhang

**Affiliations:** 1 The Second Clinical College of Guangzhou University of Chinese Medicine, Guangzhou, China; 2 Department of Oncology, The Second Affiliated Hospital of Guangzhou University of Chinese Medicine, Guangdong Provincial Hospital of Traditional Chinese Medicine, Guangzhou, China; 3 Nuclear Medicine Department, The Second Affiliated Hospital of Guangzhou University of Chinese Medicine, Guangdong Provincial Hospital of Traditional Chinese Medicine, Guangzhou, China

**Keywords:** NSCLC, ADC, *HER2*, exon 20 insertion, trastuzumab deruxtecan (T-DXd), perioperative, targeted therapy

## Abstract

**Background:**

Lung adenocarcinoma with *HER2* exon 20 insertions represents a rare subset of non-small cell lung cancer (NSCLC) associated with aggressive behavior and limited treatment options.

**Case description:**

This case report highlights the first perioperative use of trastuzumab deruxtecan (T-DXd) in a 51-year-old male diagnosed with *HER2* exon 20-mutant pulmonary adenocarcinoma (T1cN2M0). At diagnosis, a 2.2 × 2.8 × 1.5 cm lesion in the right upper lobe showed moderately elevated metabolic activity (SUVmax 4.20). An initial cycle of chemoimmunotherapy failed to achieve significant tumor reduction. Genetic testing revealed *HER2* exon 20 and *TP53* mutations, prompting a shift to two cycles of neoadjuvant T-DXd, which reduced the tumor size to 2.0 × 2.4 × 1.0 cm with substantially lowered metabolic activity (SUVmax 1.38). The lesion was successfully resected, confirming invasive adenocarcinoma with 30% viable tumor tissue and 70% stromal tissue, indicating partial tumor suppression. The patient received 14 cycles of adjuvant T-DXd, achieving 28 months of sustained remission. Molecular residual disease (MRD) monitoring through circulating tumor DNA (ctDNA) was conducted throughout adjuvant therapy, initially showing no detectable disease. However, ctDNA positivity with *HER2* and *TP53* mutations emerged later, prompting timely treatment adjustments.

**Conclusion:**

This case shows that perioperative treatment with T-DXd could induce tumor regression and sustain remission, while MRD monitoring enables early detection of molecular progression. These findings emphasize the potential of combining T-DXd and MRD monitoring to tailor treatment to improve outcomes in *HER2*-mutant NSCLC.

## 1 Introduction

Lung cancer remains the leading cause of cancer-related mortality worldwide, with lung adenocarcinoma being its most common subtype, with its treatments being continuously improved based on recent clinical trials (Bray et al., 2024). However, the management of patients with rare genetic mutations, such as human epidermal receptor 2 (*HER2*) mutations, remains challenging due to limited clinical evidence and the absence of standardized treatment guidelines. Currently, though chemotherapy in combination with immune therapy has become the standard of care for operable lung cancer, evidence for the efficacy of the combination therapy is lacking in *HER2-*mutated patients and it remains unknown whether the combination therapy has better efficacy than chemotherapy alone. Particularly, for rare mutations such as *HER2* exon 20 insertions, available perioperative data remain limited (Rossi and Di Maio, 2016). Thus, there is a growing need to explore novel therapeutic approaches that may improve surgical and oncologic outcomes in this rare subset of lung adenocarcinoma.

*HER2* mutations, including exon 20 insertions, are identified in approximately 2%–4% of non-small cell lung cancer (NSCLC) cases (Sentana-Lledo et al., 2023) and are often associated with aggressive tumor behavior and poor prognosis (Choudhary et al., 2024) as they confer insensitivity to traditional tyrosine kinase inhibitors (TKIs), highlighting the need for specific targeted therapies. However, lung cancer harboring G778_P780 variant seems to be sensitive to afatinib (Fang et al., 2020; Zhao et al., 2020) and amivantamab and zipalertinib are active drugs in this setting. Trastuzumab deruxtecan (T-DXd), a *HER2*-directed antibody-drug conjugate, has demonstrated promising efficacy in later-line treatment and has been approved for *HER2*-mutant advanced NSCLC (Goto et al., 2023; Cheng et al., 2024). Available evidence supports adjuvant or neoadjuvant targeted therapy for patients with lung cancer harboring *EGFR*-mutations or *ALK* fusions in the perioperative setting. Meanwhile, such evidence is lacking for surgically resectable NSCLC, which represents a major evidence gap in current lung cancer management, especially for patients carrying *HER2* exon 20 insertion mutations that may respond differently to treatment. Therefore, addressing this gap is of particular importance, as effective perioperative therapy could enhance tumor downstaging, facilitate complete resection, and improve long-term outcomes in these patients.

T-DXd is an antibody-drug conjugate that combines trastuzumab, a monoclonal antibody targeting *HER2*, with a topoisomerase I inhibitor via a cleavable linker for precise delivery of cytotoxic agents to *HER2*-expressing cancer cells (Nakada et al., 2019). This unique mechanism has led to remarkable efficacy in reducing tumor burden and improved survival across various cancers. Herein, we present a case report that describes the first documented use of T-DXd in a perioperative regimen for a patient with *HER2* exon 20 insertion-containing lung adenocarcinoma, which provides preliminary clinical evidence supporting the potential of T-DXd as a neoadjuvant and adjuvant therapy, where it provided notable tumor regression before surgery and contributed to durable postoperative remission.

## 2 Case presentation

**FIGURE 1:**
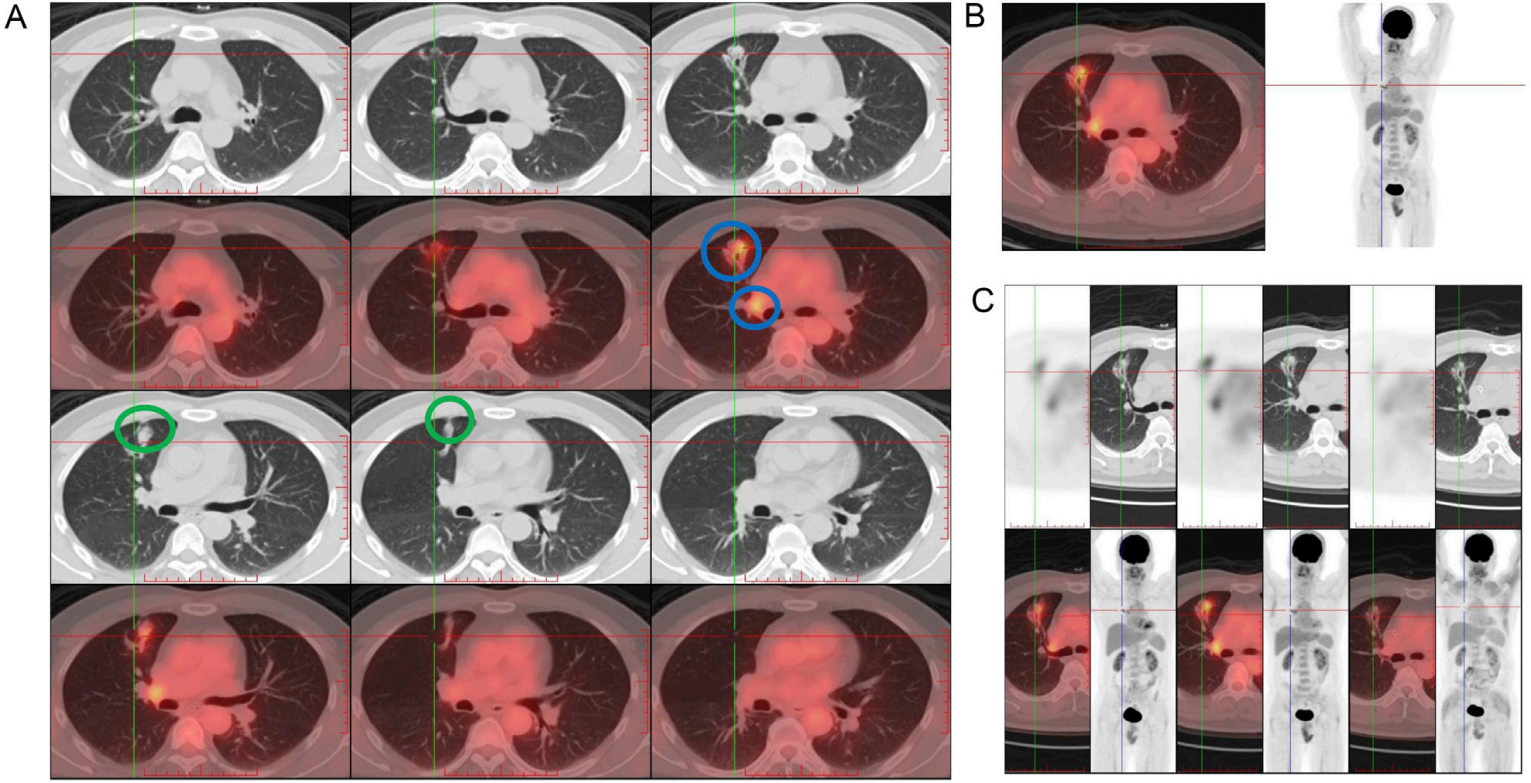
Initial imaging findings at diagnosis in January 2022. **(A)** Positron emission tomography-computed tomography (PET-CT) scan at the time of diagnosis in January 2022 revealed an irregular mass and involved lymph node (blue circles) in the anterior segment of the right upper lobe, raising suspicion of malignancy and adhesions to the chest wall and the interlobar pleura (green circle). **(B)** PET-CT scan showed moderately elevated and heterogeneous metabolic activity of the lesion and mildly hypermetabolic right hilar lymph nodes consistent with nodal involvement (left panel) and potential adhesion of the lesion to the anterior chest wall and to the interlobar pleura along the right horizontal fissure, with tractional deformation of the interlobar pleura. The coronal view is shown in the right panel. **(C)** After two cycles of T-DXd treatment, PET-CT scan in April 2022 showed a significant reduction in the size of the right upper lobe lesion.

In January 2022, a 51-year-old male (smoker, 3–5 cigarettes/day) with a history of untreated mild fatty liver, high cholesterol, and hyperuricemia underwent a routine physical examination. A chest positron emission tomography-computed tomography (PET-CT) scan revealed an irregular mass in the anterior segment of the right upper lobe and adhesions to the chest wall and the interlobar pleura, raising suspicion of lung cancer (Figure 1A). A^18^F-FDG PET-CT scan confirmed the lesion to be moderately elevated with uneven metabolic activity (Figure 1B), showing adherence to the pleura and chest wall with associated deformation, suggesting malignancy. Fibrobronchoscopic biopsy confirmed lung adenocarcinoma with lymph node metastasis (4R/11R), with peripheral blood genetic testing showing no *EGFR* mutations by amplification refractory mutation system (ARMS) method. Immunohistochemistry was positive for cytokeratin (CK) 7, napsin A, and thyroid transcription factor-1 (TTF-1), and negative for CK20, P40, and programmed death-1 (PD-1), with programmed death-ligand 1 (PD-L1) expression at 1%. The tumor tissue was negative for mucicarmine, indicating the absence of mucin production. Endobronchial ultrasound-guided transbronchial needle aspiration (EBUS-TBNA) was performed at stations 4R and 11R, with histopathologic analysis confirming metastatic carcinoma. Based on these, the patient was staged as IIIA (post-neoadjuvant pathological tumor stage T1cN2M0).

**FIGURE 2:**
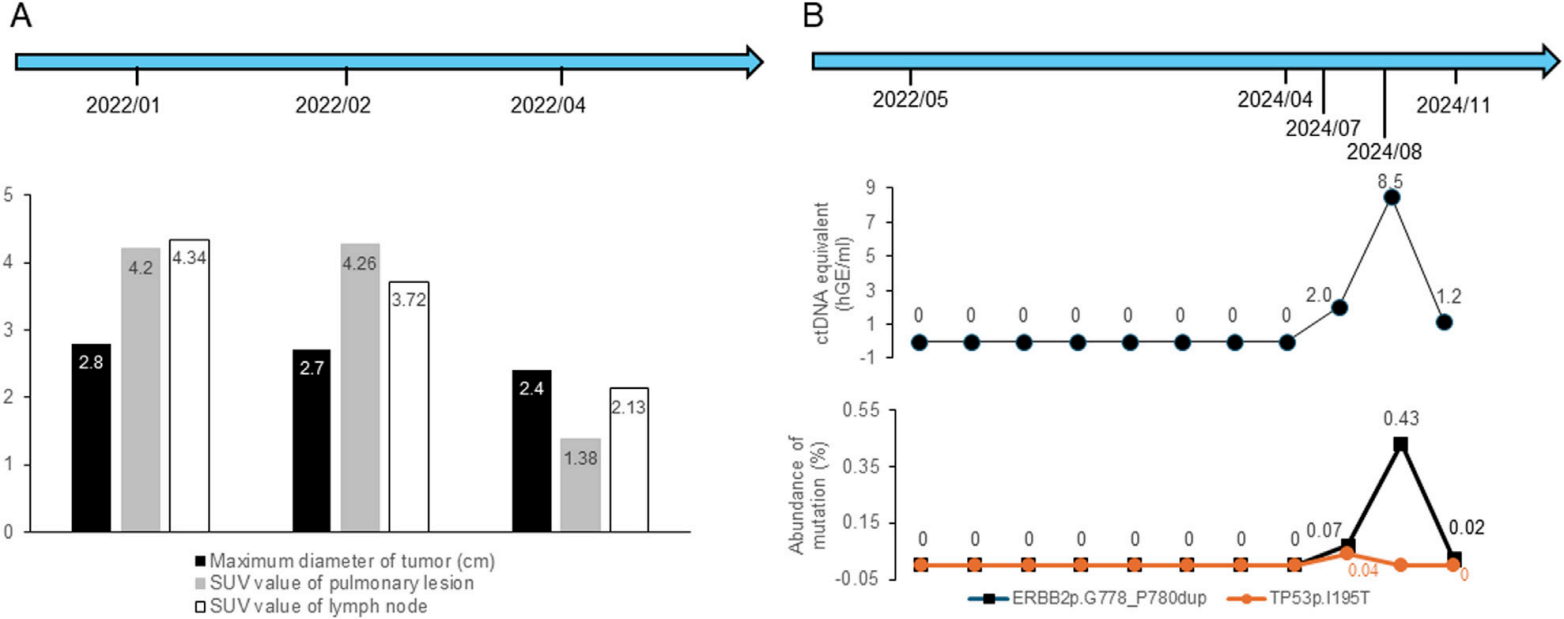
Changes in imaging parameters and ctDNA results during the treatments and follow-up periods. **(A)** Changes in maximum tumor diameter and SUV values of lung lesions and lymph nodes. **(B)** Changes in ctDNA concentration and mutation abundance. “ctDNA equivalent (hGE/mL)” refers to the total concentration of circulating tumor-derived DNA fragments quantified by digital PCR, expressed as haploid genome equivalents per milliliter of plasma.

In early February 2022, while waiting for the genetic test results of tissue samples, the patient was prescribed one cycle of pembrolizumab (200 mg, intravenous), bevacizumab (500 mg, intravenous), pemetrexed (0.9 g, intravenous), and carboplatin (600 mg, intravenous) following a multidisciplinary treatment board. After 2 weeks, the lesion size showed no significant decrease under a follow-up PET-CT. The right hilar nodes maintained their size but showed a reduced SUVmax of 3.72, while the mediastinal nodes (4R) remained 0.6 cm with a lower SUVmax of 2.32, and the 2R nodes measured 0.8 cm with an SUVmax of 3.10. Subsequently, next-generation sequencing (NGS) revealed the presence of a non-frameshift insertion mutation, p.G778-P780dup, in exon 20 of the *ERBB2* gene, with a variant allele frequency of 8.6%. Additionally, a TP53 p.I195T missense mutation in exon 6 was identified, with an abundance of 0.9%. The ARMS method with tissue samples also detected a mutation in *HER2* exon 20. Simultaneously conducted peripheral blood NGS analysis identified an ERBB2 p.G778-P780dup non-frameshift insertion mutation in exon 20, with a lower abundance of 0.4%, and a tet methylcytosine dioxygenase 2 (TET2) p.F1104Lfs*2 frameshift mutation in exon 3. Based on these findings, the treatment plan was modified, and at the end of February and mid -March 2022, he was treated with two cycles of T-DXd (400 mg, intravenous). After the initial two cycles of neoadjuvant therapy (including 1 cycle of T-Dxd), a chest CT scan indicated stability of the right upper lobe lesion compared to earlier scans, while identifying a small ground-glass nodule in the left upper lobe, raising suspicion of a preinvasive lesion and warranting close monitoring. In April 2022 (after two cycles of T-DXd), PET-CT scan revealed a significant reduction in the size of the right upper lobe lesion to 2.0 cm × 2.4 cm × 1.0 cm from 2.2 cm × 2.7 cm × 1.5 cm on previous scan, with markedly reduced metabolic activity (SUVmax: 1.38, previously 4.26) (Figure 1C). The right hilar nodes decreased to 0.6 cm (SUVmax: 2.13; Figure 2A), and the mediastinal nodes were reduced to 0.4 cm (SUVmax: 1.69) in the 4R region and 0.5 cm (SUVmax: 1.66) in the 2R region.

Although multistation N2 involvement is generally considered a relative contraindication for surgical resection under most guidelines, the patient showed marked tumor and nodal regression on PET-CT and strongly requested surgical treatment after multidisciplinary discussions, and the patient underwent radical right upper lobectomy under general anesthesia via 3D single-port thoracoscopy in April 2022 at another center., and postoperative pathology showed that 30% of the tumor tissue remained viable, while 70% was stromal tissue without necrosis, indicating tumor suppression but no downstaging. In addition, it confirmed invasive adenocarcinoma with complex glandular structures. Overall, the tumor measured 2.5 cm in diameter and was classified as grade 3 (poorly differentiated) based on the World Health Organization (WHO) classification of invasive lung adenocarcinomas. Immunohistochemical analysis revealed that the tumor tissue was positive for CK7 and TTF-1 and negative for P40, with no mucin production, suggesting that the cancer had not penetrated beyond the elastic layer of the visceral pleura (PL0), as well as absence of vascular emboli and nervous involvement. Lymph node involvement was observed in the 2R, 4R, and 10R lymph nodes. From May 2022 to September 2023, the patient received 14 cycles of adjuvant T-DXd therapy. Concurrently, eight circulating tumor DNA (ctDNA) samples were obtained for monitoring molecular residual disease (MRD) between May 2022 and April 2024, all of which yielded no detectable recurrence. In addition, a chest CT scan in February 2024 revealed no recurrence, and a cranial magnetic resonance imaging (MRI) in March 2024 showed no signs of distant metastasis. However, in July 2024, MRD monitoring identified a ctDNA concentration of 2 hGE/mL, with detectable *HER2* and *TP53* mutations. Whole body PET-MRI and chest PET-CT scan on 18 July 2024 indicated lymph node involvement, and MRD monitoring in August 2024 revealed a ctDNA concentration of 8.5 hGE/mL. The patient underwent volumetric modulated arc therapy (VMAT) until October 2024 (GTV 66 Gy/30 F, CTV 54 Gy/30 F). and consequently, the patient was treated with two cycles of albumin-bound paclitaxel plus cisplatin during radiotherapy in August and September 2024. A subsequent MRD test in November 2024 showed a ctDNA concentration of 1.2 hGE/mL (Figure 2). Treatment was switched to T-DXd, which was administered for two cycles in December 2024, and the patient discontinued treatment in May 2025.

During treatment, he experienced grade 2 nausea, vomiting, and transient elevations in alanine amino transferase (ALT; peak: 81 U/L) and γ-glutamyl transferase (γ-GT; peak: 80 U/L), which resolved without medication or dose adjustments. γ-GT peaked in February 2022 and ALT peaked in December 2022. Blood tests and cardiac assessments, including ECG till April 2024, remained normal.

## 3 Discussion

This case demonstrates significant tumor regression with neoadjuvant and adjuvant T-DXd therapy. After the initial immunotherapy and chemotherapy could not significantly reduce the tumor size and metabolic activity, two cycles of neoadjuvant T-DXd were given, which resulted in a promising decrease in metabolic activity. Postoperative adjuvant T-DXd maintained these effects, with no recurrence over 28 months. Although T-DXd is currently approved for *HER2*-mutant lung cancer in the later-line setting (CSCO, 2024), this case highlights its potential in perioperative management for *HER2* exon 20 insertion-mutant NSCLC, an aggressive subtype. The favorable tumor response contributed to successful, complication-free surgical resection.

Until now, no major oncology guidelines recommend using T-DXd in the neoadjuvant or adjuvant settings for *HER2*-mutant NSCLC (CSCO, 2024; NCCN, 2024), and its perioperative use remains investigational. T-DXd was approved in China in October 2024 for *HER2*-mutant NSCLC, marking a significant step forward in expanding treatment options for this challenging subgroup beyond traditional chemotherapy and non-targeted approaches. Before T-DXd approval, advanced *HER2*-mutant NSCLC was often treated using protocols similar to those for driver gene-negative cases, relying primarily on standard chemotherapy with/without immunotherapy or anti-angiogenic drugs due to the absence of targeted therapies specific to *HER2* mutations (Tsuboi et al., 2023; Wu et al., 2024). The 2021 Chinese Society of Clinical Oncology (CSCO) guidelines provided a Grade III recommendation for pyrotinib, a small-molecule tyrosine kinase inhibitor, as a later-line treatment for *HER2*-mutant advanced NSCLC (Wang et al., 2022).

Historically, neoadjuvant chemotherapy for NSCLC has demonstrated only moderate efficacy, with limited long-term survival benefits (Forde et al., 2022) while the evidence for the efficacy of chemoimmunotherapy, the current standard of care, remains scant in *HER2-*mutated patients. In our case, the response observed suggests that T-DXd can achieve meaningful tumor regression, allowing complete resection. The ADAURA (Tsuboi et al., 2023) and ALINA (Wu et al., 2024) trials represent important references for the case discussed, as they highlighted the transformative potential of integrating targeted therapies into postoperative management of oncogenic-driven NSCLC. Although these trials focused on *EGFR* and *ALK* alterations, they underscore the importance of mutation-specific adjuvant treatment, a principle applicable to *HER2*-mutant disease as well.

T-DXd was prioritized over other *HER2*-targeted agents because its antibody-drug conjugate mechanism enables direct delivery of a topoisomerase I inhibitor to *HER2*-expressing tumor cells and exerts a bystander effect that enhances activity even in tumors with heterogeneous *HER2* expression. Small-molecule TKIs or monoclonal antibodies have shown limited activities in lung cancer whereas evidence from the DESTINY-Lung02 and related studies demonstrated efficacy and sustained disease control with T-DXd (Goto et al., 2023), providing the rationale for selecting T-DXd. These mechanisms may also explain the marked stromal tissue replacement (70%) post-treatment, suggesting extensive tumor cell clearance and fibroinflammatory remodeling, which are consistent with antibody-drug conjugate activity.

For our case, T-DXd was chosen due to the presence of an ERBB2 p.G778-P780dup non-frameshift insertion mutation in exon 20, often resistant to classical EGFR TKIs (Zeng et al., 2021), thus, making targeted *HER2* inhibitors more suitable. In addition, it was approved as a Grade III recommendation for *HER2*-mutant NSCLC treatment in China and was recently elevated to a Grade II recommendation for the same indication, though not in the adjuvant setting (Yingge et al., 2023; CSCO, 2024). With an abundance of 8.6%, the ERBB2 mutation represented a significant driver of the tumor’s behavior, highlighting the potential efficacy of T-DXd. Furthermore, the co-occurrence of a TP53 p.I195T missense mutation in exon 6, although present at a lower abundance (0.9%) and may lead to a more aggressive tumor phenotype and poorer prognosis (Kelemen et al., 2018), our patient demonstrated robust antitumor activity with T-DXd, possibly due to its mechanism of delivering a potent cytotoxic payload directly to *HER2*-expressing cells and its unique bystander effect that improves efficacy even in heterogeneous tumors (Yver et al., 2020). While the integration of targeted therapies like T-DXd in the adjuvant setting aligns with the broader trend of tailoring treatment to specific oncogenic drivers, these advances highlight the need for further research and large-scale clinical trials for validation.

The recurrence observed in this case may be attributed to the aggressive nature of *HER2* exon 20-mutant NSCLC, characterized by tumor heterogeneity and therapy resistance, compounded by co-occurring TP53 mutations contributing to genomic instability. Moreover, preoperative regional lymph node metastasis and the residual viable tumor tissue (30%) post-surgery might have also be contributors for recurrence (Dickhoff et al., 2016), potentially due to inadequate neoadjuvant treatment. However, it should be noted that the optimal duration of adjuvant therapy remains an area of investigation as there is limited evidence on the appropriate length of anti-*HER2* therapy in the perioperative setting. In addition, caution is advised in interpreting the results of this single case report, which need to be validated in multicenter cohort studies. In this case, 14 cycles of T-DXd were administered over 16 months, followed by a switch to salvage therapy upon recurrence. The ctDNA-based MRD monitoring successfully revealed potential micrometastatic disease (Xue et al., 2024), which was undetectable by imaging, thereby offering a significant advantage by detecting molecular progression earlier than imaging, enabling timely interventions and even allowing therapy de-escalation when MRD returned to negative, improving patient outcomes and reducing toxicity. However, further research is required to standardize the duration of adjuvant therapy and the optimal use of MRD status, which could help improve outcomes while minimizing unnecessary treatment, especially for aggressive cancer.

## 4 Conclusion

This case highlights T-DXd’s efficacy in treating *HER2* exon 20-mutant NSCLC, showing that it achieved significant tumor regression that allowed for complete surgical resection and long-term remission. In addition, MRD monitoring was valuable for the early detection of molecular progression and facilitated timely therapeutic intervention. Further studies are warranted to validate the use of T-DXd in perioperative settings and to optimize MRD-guided management strategies for this aggressive subtype.

## Data Availability

The original contributions presented in the study are included in the article/supplementary material, further inquiries can be directed to the corresponding authors.

## Abbreviations

ALT, alanine aminotransferase; ARMs, amplification-refractory mutation system; CK, cytokeratin; CK7, cytokeratin 7; CK20, cytokeratin 20; CSCO, Chinese Society of Clinical Oncology; ctDNA: circulating tumor DNA; ECG, electrocardiogram; EGFR, epidermal growth factor receptor; ERBB2, erb-b2 receptor tyrosine kinase 2; GGT, gamma-glutamyl transferase; *HER2*, human epidermal growth factor receptor 2; MRD, minimal residual disease; MRI, magnetic resonance imaging; NGS, next-generation sequencing; NSCLC, non-small cell lung cancer; PET-CT, positron emission tomography-computed tomography; PD-1, programmed death-1; PD-L1, programmed death-ligand 1; PL0, pleural invasion status 0; T-DXd, trastuzumab deruxtecan; TET2, tet methylcytosine dioxygenase 2; TTF-1, thyroid transcription factor-1; WHO, World Health Organization
